# Supplementary material for: Role of Hibernation Promoting Factor in Ribosomal Protein Stability during Pseudomonas aeruginosa Dormancy
Source: Int J Mol Sci. 2020 Dec 14;21(24):9494. doi: 10.3390/ijms21249494 (PMC7764885; doi:10.3390/ijms21249494)
Supplement: Supplementary file 1 [file ijms-21-09494-s001.pdf]

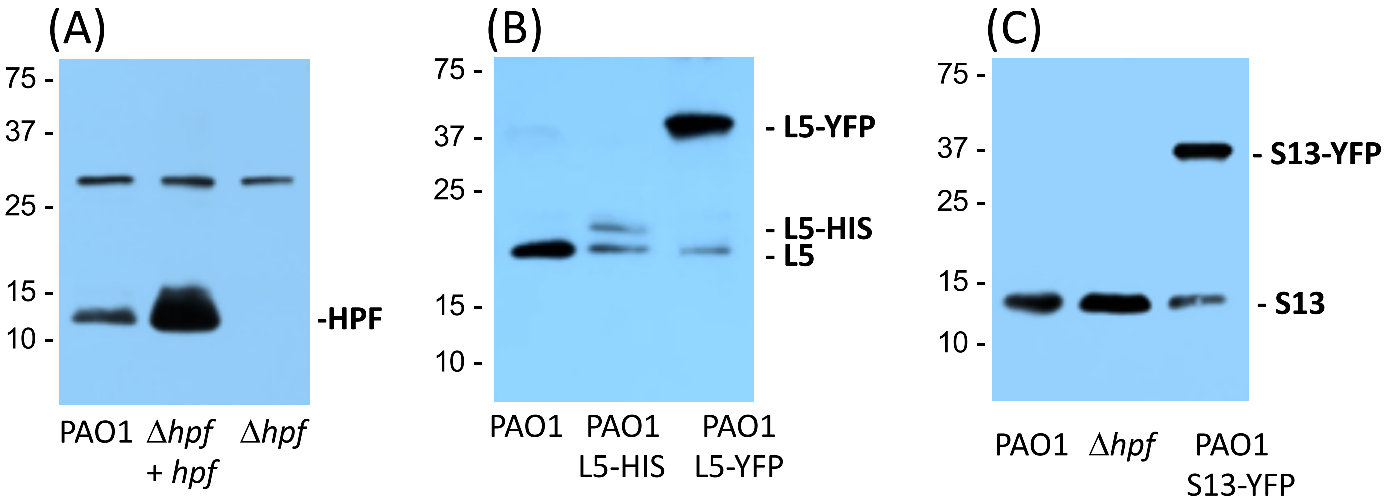


Figure S1. Immunoblotting showing efficacy of antibodies to *P. aeruginosa* HPF, S13 and L5. (A) Antibodies to HPF showing a band at 11.6 kDa for *P. aeruginosa* PAO1 and for *P. aeruginosa Δhpf* + *hpf*, but not for *P. aeruginosa Δhpf*. A cross-reactive band is present at 30 kDa. (B) Antibodies against L5, showing a band at 20 kDa for the *P. aeruginosa* L5 protein, a band at 21 kDa for the L5-6xHis fusion protein, and a band at 48 kDa at the L5-YFP fusion protein. (C) Antibodies to S13 showing a 13 kDa band for the *P. aeruginosa* S13 protein and a 41 kDa band against the S13-YFP fusion protein.

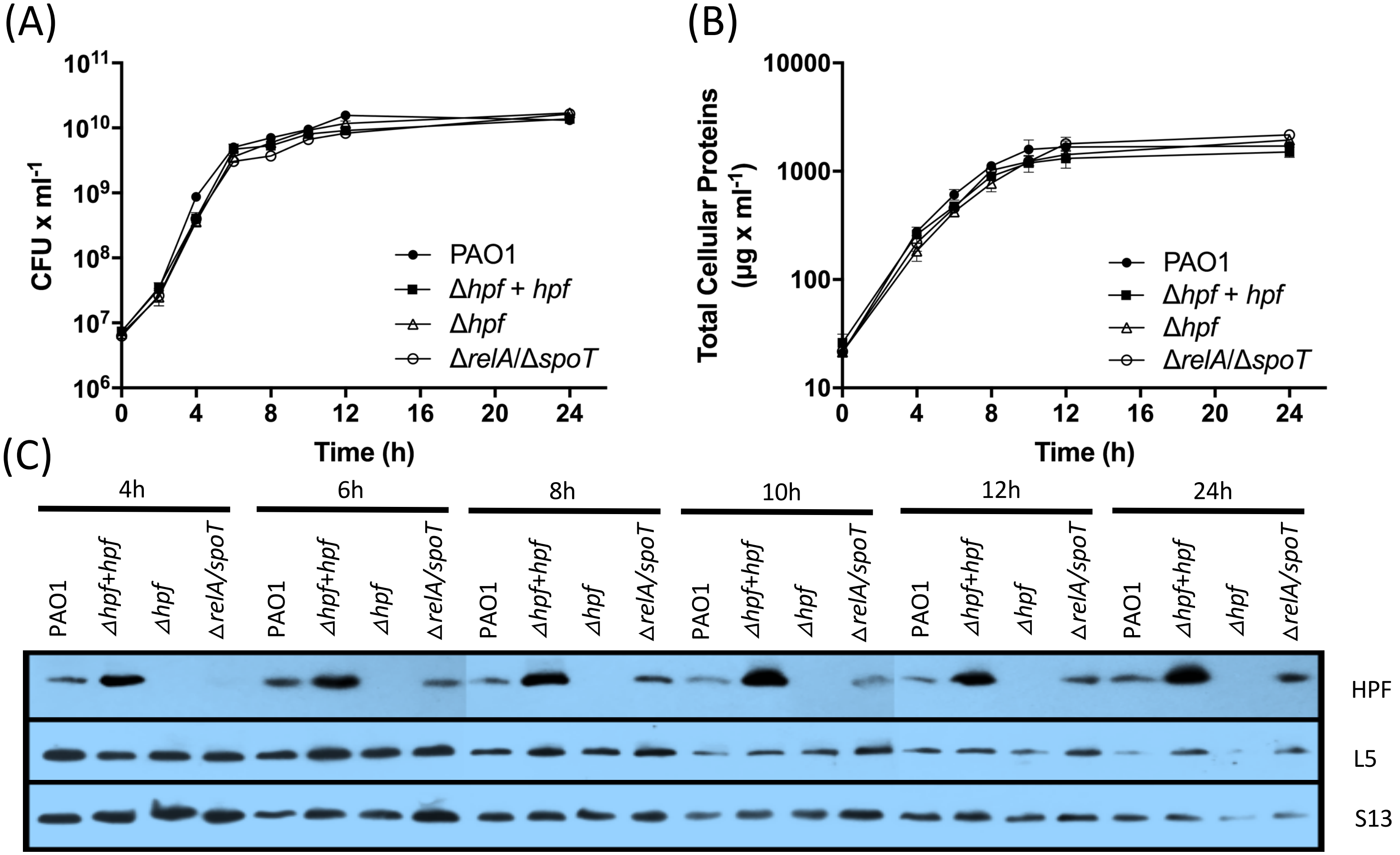


Figure S2. Growth curve and protein abundances of *P. aeruginosa.* (A) Growth of *P. aeruginosa* PAO1, Δ*hpf*, Δ*hpf* +*hpf*, and Δ*relA*/Δ*spoT* assayed as colony forming units (CFUs) over time. (B) Growth of strains quantified as total cellular protein over time. (C) Representative Immunoblots analysis of HPF, L5 and S13 over the course of growth for *P. aeruginosa* PAO1 and *P. aeruginosa* strains Δ*hpf*, Δ*hpf* +*hpf*, and Δ*relA*/Δ*spoT* . (A) and (B) show the mean and standard error of the mean for three replicates.

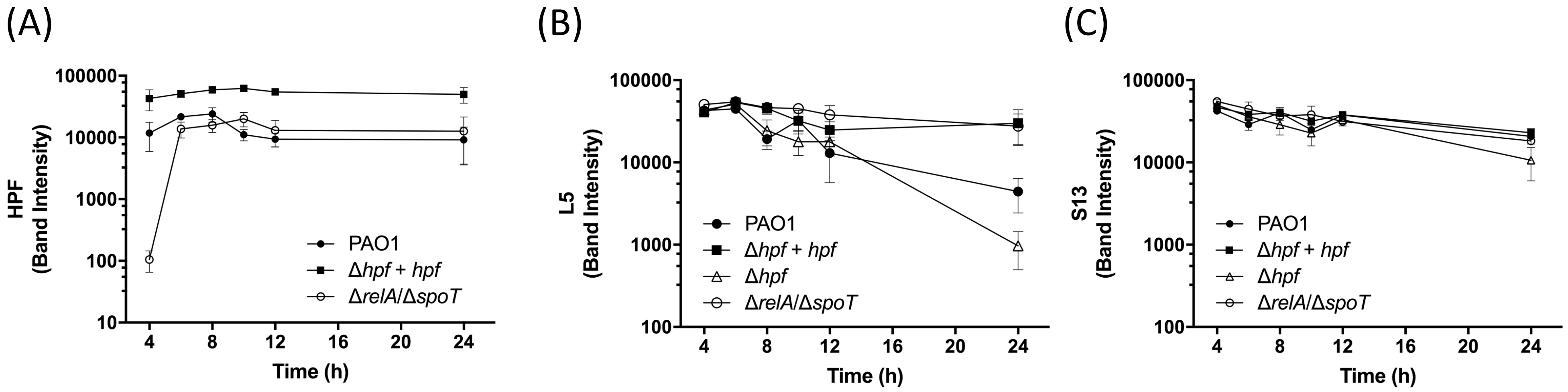


Figure S3. Proteins HPF, L5, and S13 over the course of cell growth shown in Fig S2, quantified from immunoblots by densitometry. Protein abundances were nomalized by loading equivalent amounts of total cellular protein (15 ug) per lane, and the average signal intensity was obtained from three replicates. (A) Quantification of HPF over the course of a 24 h growth curve. (B) Quantification of L5. (C) Quantification of S13. Error bars show the standard error of the mean for three independent biological replicates.

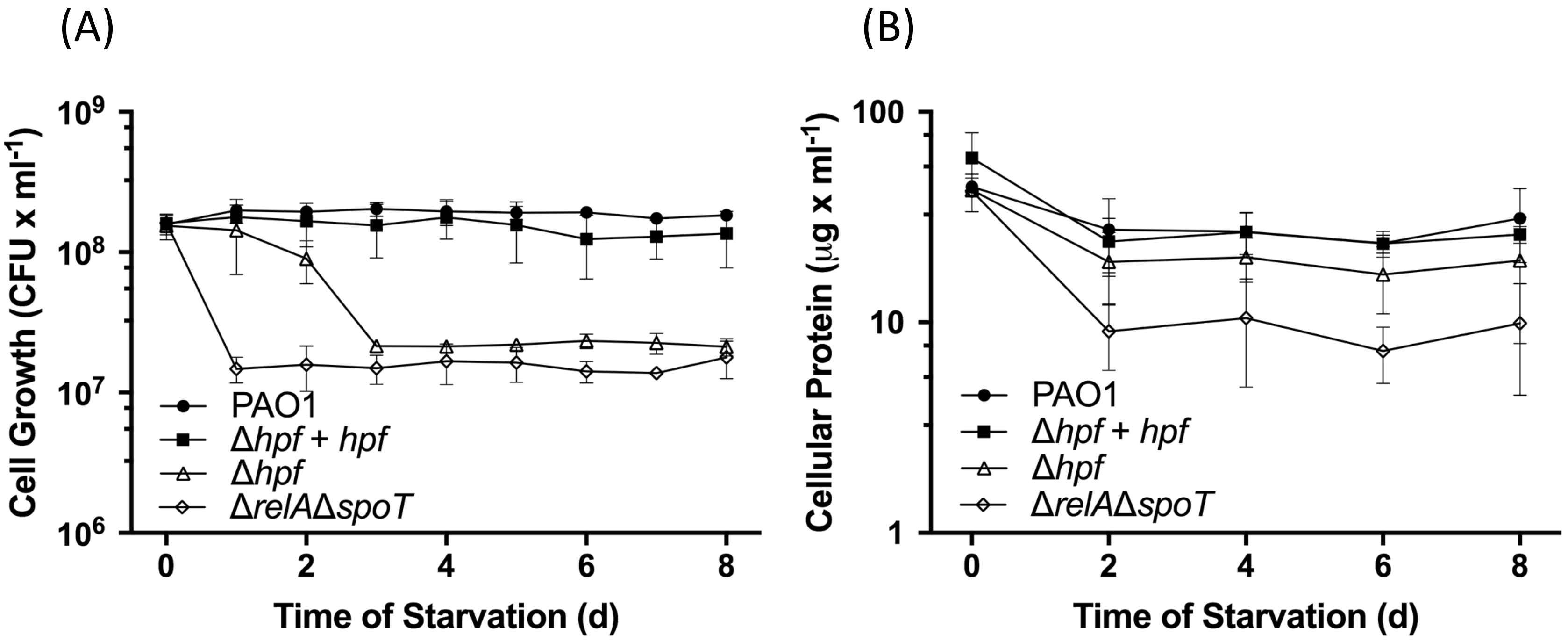


Figure S4. (A) Resuscitation of *P. aeruginosa* and mutant derivatives over the course of starvation in PBS at 37C, following growth in TSB to $O.D._{600}$ = 1.0. (B) Total cellular protein of *P. aeruginosa* strains from panel A. Shown are the mean and standard deviation of three replicates.

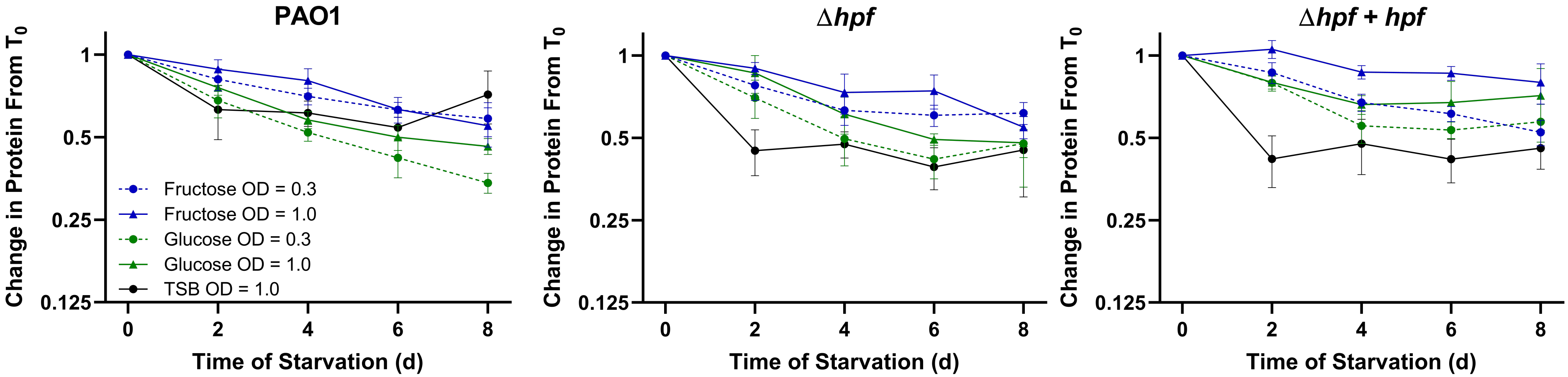


Figure S5. Cellular protein abundances of *P. aeruginosa* and mutant derivatives over the course of starvation when cells were pre-cultured in MOPS minimal medium with fructose or glucose as the sole carbon source. (A) Total cellular proteins of *P. aeruginosa* PAO1. (B) Total cellular proteins of *P. aeruginosa* Δ*hpf*. (C) Total cellular proteins of *P. aeruginosa* Δ*hpf* + *hpf*.

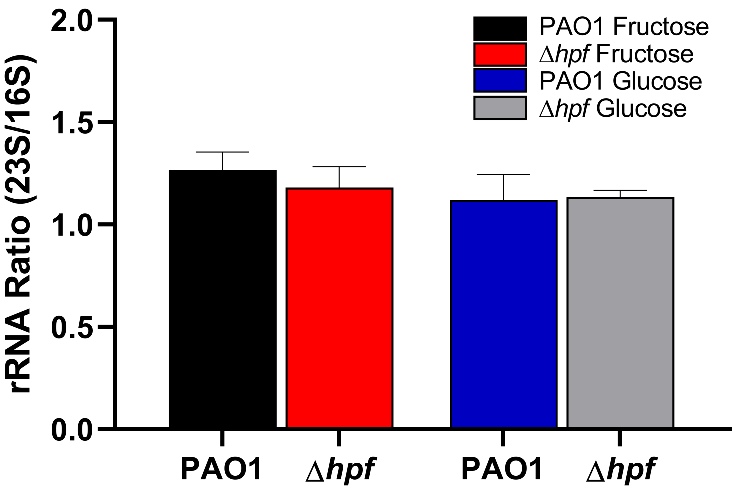


Figure S6. Ratios of 23S/16 rRNA for *P. aeruginosa* and P. aeruginosa Δ*hpf* cultured in MOPS minimal medium to O.D.600 = 1.0, prior to starvation. Shown are the mean and the standard error of the mean for three independent biological replicates.
